# Serum-Free Thyroxine Levels Were Associated with Pulmonary Hypertension and Pulmonary Artery Systolic Pressure in Euthyroid Patients with Coronary Artery Disease

**DOI:** 10.1155/2017/4832608

**Published:** 2017-06-22

**Authors:** Bingjie Wu, Jingjing Jiang, Minghui Gui, Lin Liu, Qiqige Aleteng, Shanshan Wang, Xiaojing Liu, Yan Ling, Xin Gao

**Affiliations:** Department of Endocrinology and Metabolism, Zhongshan Hospital, Fudan University, No. 180 Fenglin Road, Shanghai 200032, China

## Abstract

The aim of this study was to evaluate the association between thyroid hormone levels, pulmonary hypertension (PH), and pulmonary artery systolic pressure (PASP) in euthyroid patients with coronary artery disease (CAD). A cross-sectional study was conducted in individuals who underwent coronary angiography and were diagnosed as CAD from March 2013 to November 2013. 811 subjects (185 women and 626 men) were included in this study. PASP was measured by transthoracic Doppler echocardiography. 86 patients were diagnosed as PH and had significantly higher free thyroxine (FT_4_) levels than those without PH. Multiple logistic regression analysis demonstrated an independent association of FT_4_ levels with PH after adjustment of gender, age, body mass index, systolic blood pressure, left ventricular ejection fraction, hypertension, and medication use of calcium channel blockers, ACE inhibitors, angiotensin II receptor antagonists, and nitrates. Serum-free triiodothyronine (FT_3_) and thyroid-stimulating hormone (TSH) were not associated with PH. Furthermore, multivariate linear regression analysis showed that FT_4_ levels emerged as an independent predictor for PASP, while FT_3_ and TSH levels were not associated with PASP. Our study demonstrated that, in euthyroid patients with CAD, FT_4_ was an independent risk factor for PH, and FT_4_ levels were independently associated with PASP.

## 1. Introduction

Pulmonary hypertension (PH) is a hemodynamic and pathological state with a high rate of mortality that may lead to right heart failure and ultimately death if untreated. PH can be found in multiple clinical conditions and its symptoms are nonspecific. Badesch et al. [1] brought attention to the relationship between PH and hypothyroidism and growing evidences confirmed the association between PH and thyroid diseases thereafter. Curnock et al. [2] revealed in their retrospective study that the prevalence of hypothyroidism in 41 patients with PH was 22.5%. Chu et al. [3] found that the prevalence of autoimmune thyroid disease in the patients with PH was 49%. In another study, the prevalence of thyroid disease was 24% in PH patients and 15% in the control group [4]. On the other hand, patients with thyroid disease, predominantly hyperthyroidism, also have higher pulmonary arterial pressure than healthy subjects. The prevalence of PH among patients with hyperthyroidism was reported to be 34–65% [5–9], and pulmonary artery systolic pressure (PASP) decreased after treatment of hyperthyroidism.

These results aforementioned suggested a close relationship between thyroid diseases and PH. Thyroid hormones may play an important role in regulating PASP. However, the association of thyroid hormones with PH remains controversial. Marvisi et al. [10] demonstrated that in population with recently diagnosed hyperthyroidism without antithyroid treatment, PASP was associated with thyroid-stimulating hormone (TSH) and free thyroxine (FT_4_) levels. However, Sugiura et al. [11] showed that PASP was not significantly correlated with free triiodothyronine (FT_3_) or FT_4_ in patients with Graves' disease. And in patients with Hashimoto's thyroiditis, it was shown that FT_3_, FT_4_, or TSH was not independently related to PASP [12, 13].

Meanwhile, PASP is considered as an important prognostic factor for assessing morbidity and mortality in patients with CAD [14–16]; thus, it is essential to find out the potential risk factors for PH in CAD patients. According to previous studies, it is acknowledged that thyroid dysfunction could induce PH via multiple pathways, but whether thyroid hormones within reference range would affect PASP is still uncertain. To the best of our knowledge, the association between thyroid hormones and PH was never examined in CAD subjects. To address this issue, we sought to clarify the possible relationship between thyroid hormones, PH, and PASP in euthyroid patients with CAD.

## 2. Study Population and Methods

During March 2013 to November 2013, we consecutively enrolled 2045 patients who were admitted to the Department of Cardiology of Zhongshan Hospital for suspected CAD and underwent coronary angiography. Data of participants were collected by a structured interview and medical record review. Smoking, history of hypertension, heart failure, chronic obstructive pulmonary disease, and medication use (calcium channel blockers, ACE inhibitors, angiotensin II receptor antagonists, and nitrate drugs) were recorded. Coronary angiography was performed by experienced doctors using a digital angiography system (AXIOM Artis dFC, Siemens, Germany). Coronary artery branches including the left coronary artery, left anterior descending artery, left circumflex artery, left marginal artery, diagonal branch, right coronary artery, posterior descending artery, and right marginal artery were evaluated. A luminal stenosis of 50% or more of any branch was defined as CAD.

Exclusion criteria were listed as follows: history of heart failure, chronic obstructive pulmonary disease, hypothyroidism, hyperthyroidism, severe systemic diseases, malignancy, using medications (antithyroid medications, thyroid hormone, amiodarone, and glucocorticoid hormone) influencing thyroid function, luminal stenosis of all branches less than 50% measured by coronary angiography, and patients with uncompleted data. Finally, 811 subjects (185 women and 626 men) were included in the analyses. The study was approved by the Ethical Committee of Zhongshan Hospital affiliated to Fudan University. Informed consent was obtained from each participant.

Anthropometric parameters were collected for each individual. Weight and height were measured and body mass index (BMI) was calculated as weight divided by height squared (kg/m^2^). Blood samples were obtained before angiography. Serum FT_3_, FT_4_, and TSH were measured by a model 7600 automated bioanalyzer (Hitachi, Tokyo, Japan). Hypothyroidism was defined as TSH > 4.2 mU/L with or without decreased thyroid hormones, and hyperthyroidism was defined as TSH< 0.27 mU/L with or without elevated thyroid hormones.

Transthoracic Doppler echocardiogram was performed using an iE33 Echocardiograph (Philips Medical Systems, Bothell, WA, USA). Two-dimensional images were obtained. The end-diastolic volume (EDV) and end-systolic volume (ESV) were measured, then left ventricular ejection fraction (LVEF) was calculated as follow: LVEF (%) = (EDV − ESV)/EDV × 100%. The tricuspid regurgitation peak velocity (TRPV) and right atrial pressure (RAP) were recorded, and PASP was calculated using the formula: PASP = 4 × TRPV + RAP. In addition, 20 participants were selected randomly to assess the interobserver and intraobserver variability. Two experienced echocardiographers performed the exact same procedure of echocardiogram to determine the interobserver variability. One of the echocardiographers repeated the procedure the next day to determine the intraobserver variability. The interobserver variability coefficients were 5.1%, 4.5%, 5.6%, 6.1%, 5.9%, and 7.9% for EDV, ESV, LVEF, TRPV, RAP, and PASP, respectively. The intraobserver variability coefficients were 4.2%, 4.3%, 5.0%, 4.9%, 5.7%, and 7.0% for EDV, ESV, LVEF, TRPV, RAP, and PASP, respectively. PH was defined as PASP >40 mmHg [17].

## 3. Statistical Analyses

Continuous variables were reported as mean ± standard deviation, while categorical variables were represented by frequency and percentage. To determine the potential factors influencing PH, participants were stratified according to the values of PASP, and the stratifications were defined as follows: non-PH: PASP ≤ 40 mmHg, mild PH: 40 mmHg < PASP ≤ 50 mmHg, and moderate-to-severe PH: PASP> 50 mmHg [18]. On the other hand, in order to compare the differences of PASP levels between different thyroid functions, the study subjects were also stratified according to the tertile groups of thyroid hormones. FT_3_ tertiles are divided as follows: tertile 1: FT_3_ ≤ 4.1 (*n* = 308), tertile 2: 4.1 < FT_3_ ≤ 4.5 (*n* = 236), and tertile 3: FT_3_> 4.5(*n* = 267); FT_4_ tertiles: tertile 1: FT_4_ ≤ 14.2 (*n* = 260), tertile 2: 14.2 < FT_4_ ≤ 16.1 (*n* = 279), tertile 3: FT_4_> 16.1 (*n* = 272); and TSH tertiles: tertile 1: TSH ≤ 1.57 (*n* = 275), tertile 2: 1.57 < TSH ≤ 2.59 (*n* = 277), and tertile 3: TSH> 2.59 (*n* = 259). One-way ANOVA analysis and analysis of covariance were used for comparisons for continuous variables, and chi-square tests were performed for categorical variables among the groups. Logistic regression analysis was performed to determine the independent risk factors for PH. Multivariate linear regression analysis was used to determine independent risk factors influencing PASP. All statistical analyses were performed using the SPSS statistics version 17.0 (SPSS Inc., Chicago, IL). All tests were two tailed, and *P* < 0.05 was considered statistically significant.

## 4. Results

The mean age of the study subjects was 63.3 ± 9.87 years, and the mean PASP was 34 ± 8 mmHg. The study population was divided into two groups according to PASP measured by echocardiography: 86 suffering from PH and 725 had normal PASP.  Table 1 shows the baseline clinical characteristics of each group. Compared with those with the non-PH group, patients with PH were older. There were more smokers in the PH group compared to those in the non-PH group (*P* < 0.05). The PH group had significantly higher levels of FT_4_ (*P* < 0.05) and lower LVEF (*P* < 0.05) than those of the non-PH group. Serum FT_3_ and TSH did not differ significantly between the two groups.

Furthermore, we compared the thyroid hormone levels among subgroups with different PASP (Table 2). An increase of serum FT_4_ levels was found as PASP increased. FT_4_ levels of the mild PH and moderate-to-severe PH groups were higher than those in the non-PH group (15.75 ± 2.31 and 16.80 ± 2.94 versus 15.30 ± 2.07 mmol/L, both *P* < 0.05). Serum TSH and FT_3_ levels were not significantly different among subgroups with different PASP. Moreover, the study subjects were further stratified according to thyroid hormones tertiles (Table 3). In the FT_3_ tertile groups, PASP were higher in the first tertile than in the other two tertiles (*P* < 0.01). After adjusted for age, gender, BMI, and LVEF, the significant difference disappeared (*P* = 0.06). No significant difference of PASP was found among the FT_4_ and TSH tertile groups (*P* = 0.42 and *P* = 0.36, resp.).

To determine the relationship between PH and thyroid hormones, multiple logistic regression analyses were performed (Table 4). After adjustment of age, gender, BMI, smoking, LVEF, hypertension, and use of calcium channel blockers, ACE inhibitors, angiotensin II receptor antagonists, and nitrates, FT_4_ levels were independently associated with the risk of PH (OR = 1.12, 95% CI 1.01–1.25, *P* = 0.03). However, levels of serum FT_3_ and TSH were not independently associated with PH (OR = 1.18, 95% CI 0.79–1.82, *P* = 0.45 and OR = 0.97, 95% CI 0.84–1.12, *P* = 0.70, resp.).

Multivariate linear regression analyses were performed to further analyze the risk factors influencing PASP (Table 5). Serum FT_4_ levels (*β* = 0.27 ± 0.12, *P* = 0.03) and age (*β* = 0.11 ± 0.03, *P* < 0.01) were positively associated with PASP adjusted for age, gender, BMI, smoking, LVEF, hypertension, and use of calcium channel blockers, ACE inhibitors, angiotensin II receptor antagonists, and nitrates. And a negative association was observed between LVEF and PASP (−0.13 ± 0.03, *P* < 0.01). In contrast, levels of FT_3_ (*β* = −0.53 ± 0.49, *P* = 0.28) and TSH (*β* = 0.00 ± 0.15, *P* = 0.99) were not significantly associated with PASP.

## 5. Discussion

In the present study, we investigated the relationship between thyroid function and PASP in euthyroid patients with CAD. We observed that serum FT_4_ levels were not only significantly higher in patients with PH than patients without PH but also independently associated with PH and PASP. In contrast, serum FT_3_ or TSH levels were not associated with PH or PASP. To the best of our knowledge, the present study is the first to explore the independent relationship between thyroid hormones and PH in euthyroid patients with CAD.

CAD is highly prevalent worldwide and has become one of the top causes of death. Left heart diseases have been considered as a common cause of PH. It may lead to an increase in left atrial pressure, and subsequently, pulmonary artery pressure increases due to the passive backward transmission [19]. Meanwhile, PH is potential to alter right ventricular afterload, resulting in increasing cardiac output and may lead to left ventricle dysfunction [14]. A retrospective analysis performed in 6611 patients with CAD undergoing bilateral heart catheterization indicated that 61.3% had abnormal right heart catheterization values and 4.3% met formal criteria for PH [20]. It was also reported that secondary PH predicted mortality in patients with acute myocardial infarction [15]. The tricuspid regurgitation and end-diastolic pulmonary regurgitation gradients, in conjunction with PASP, have been emerged as a potential predictor of mortality and heart failure hospitalization in patients with stable coronary artery disease [16]. In this setting, it is essential to determine the possible risk factors for PASP in CAD. On the other hand, several studies demonstrated that levels of FT_4_ were independently associated with increased prevalence of coronary calcification and FT_3_ levels were independent predictors for both the presence and severity of CAD in euthyroid population [21–23]. However, whether thyroid hormone levels could be a factor for increasing PASP which predicts prognosis of patients with CAD is still uncertain.

Growing evidences suggested that thyroid disease is closely related to PH. Patients with thyroid disease had relatively high PASP values [5–9], while PH patients had high prevalence of thyroid diseases [1–4]. A number of prospective studies found that increase of PASP in hyperthyroidism is usually slight and reversible, and would normalize with the achievement of euthyroid status [5–9], which suggested thyrotoxemia, at least in part, is related to PH.

As for the pathogenetic mechanisms of PH under the background of hyperthyroidism and increased thyroid hormones levels, there are several hypotheses. Firstly, it was demonstrated that thyroid hormones may exert a direct action on pulmonary endothelial and smooth muscle cells [24–27]. Huesseini et al. reported that subtotal thyroidectomy reversed the development of severe experimental PH in SuHx rats, and the PH phenotype could be restored after a supplement of thyroxine. And they also showed that thyroid hormones promote endothelial cell proliferation through extracellular signal-regulated protein kinase 1 and 2 phosphorylation, and the expression of α_v_*β*_3_ integrin, fibroblast growth factor- (FGF-) 2, and FGF receptor. Lai et al. indicated that propylthiouracil treatment could suppress the smooth muscle cells via gamma-secretase-mediated Notch3 signaling pathway, especially presenilin enhancer 2 (Pen-2) subunit, resulted in attenuated experimental PH. Secondly, thyroid hormones also have many effects on the heart. Even in euthyroid patients, thyroid hormone levels are associated with heart rate and cardiac structure and function [28]. As a result, increased hemodynamic stress could promote an increase in endothelial shear stress of the pulmonary arteries, followed by endothelial dysfunction [29], which might be an important mechanism for the onset of PH. Thirdly, thyroid hormones enhanced catecholamine sensitivity and decreased cholinergic tone, resulting in pulmonary vasoconstriction. High levels of thyroid hormones also lead to increased metabolism of the intrinsic pulmonary vasodilating substances (prostacyclin, nitric oxide) and decreased metabolism of the vasoconstrictors (serotonin, endothelin 1, and thromboxane). The net effects of these processes contributed to an increase in pulmonary vascular resistance [30].

Former studies explored the association of thyroid hormones and PH predominantly in patients with hyperthyroidism, and the results were controversial. In an observational study by Marvisi et al. [10], correlations between PASP with both TSH and FT_4_ levels were observed in newly diagnosed hyperthyroidism patients without antithyroid treatment. Another study [11] recruited 50 consecutive patients with Graves' disease. No significant difference of FT_3_, FT_4_, or TSH levels was found between the PH and non-PH groups, and PASP was not related to FT_3_ or FT_4_ levels.

Although more and more human studies confirmed the relationship between PH and hyperthyroidism, whether thyroid hormones in the normal range affect the cardiovascular system still lacks evidence. Our study demonstrated that serum FT_4_ levels were independently associated with PH and PASP in euthyroid CAD patients, which suggested that FT_4_ in the normal range might play a role in the development of PH. However, only one previous study investigated the association between PH with FT_3_ and FT_4_ in euthyroid patients. Ciccone et al. [12] enrolled 93 euthyroid patients, 70 suffering from Hashimoto's thyroiditis and 23 controls. They demonstrated PASP were not independently related to FT_3_, FT_4_, or TSH in this population. The discrepancy of the two studies might be due to the different study populations. Our study focused on 811 CAD patients with mean age 63.3 years old, and among them, 77.2% were males. Their study was conducted in female patients with Hashimoto's thyroiditis who were older than 18 years old, and their sample size was much smaller than ours. Further studies are needed to confirm the association of thyroid hormones with PH and PASP in euthyroid subjects.

It is widely accepted that the major effects of thyroid hormones on the cardiovascular system are exerted by triiodothyronine (T_3_), which was mainly converted from the inactive form, thyroxine (T_4_), in the peripheral tissues. Considering this conversion process, FT_3_ was supposed to have a similar effect on PH as FT_4_ did. However, we did not find any relationship between PASP and FT_3_. Compared with that of FT_4_, the reference range of FT_3_ for euthyroid status was relatively narrower; and therefore, it might be underpowered to detect a statistic significant association between FT_3_ and PASP. In addition, although there is a regulatory negative feedback loop between TSH and thyroid hormones, the correlations between TSH and FT_4_ and FT_3_ were relatively low in our study (*β* = −0.17 and *β* = −0.14, resp.). As a result, our study did not find that TSH was associated with PASP. Sahin et al. [13] recruited 30 consecutive euthyroid patients with Hashimoto's thyroiditis and 30 healthy controls and found that correlation between PASP and TSH was not significant, which was consistent with our observations.

Besides, our results showed that age and smoking were independent risk factors for PH. According to previous studies, advanced age and a greater tobacco exposure were associated with severely reduced diffusing capacity of the lung for carbon monoxide in PH patients [31]. Tobacco smoke exposure can lead to an elevation of pulmonary artery pressure, long before destruction of the lung parenchyma [32–34]. Keusch et al. observed a significantly higher smoking prevalence in PH men and significantly more exposure to tobacco smoke in PH women to in a case-control study [35]. Another study in hemodialysis patients also found that PH was related to cigarette smoking [36]. In the Rotterdam Study [37], the mean age of their study subjects was 76.4 years which was similar to our population. The prevalence of PH was higher in older participants compared to that in younger ones, and older age was independently associated with higher PASP, which was in agreement with our findings. And the association of age with PH was also observed in other studies [38–41].

Our study has several limitations. First, since this was a retrospective study, the causative relationship between FT_4_ and PASP cannot be determined. And lacking of data of some patients may contribute to statistic bias in our analysis. Prospective studies are needed to confirm our results in the future. Second, the use of medical records might be a further limitation that memory bias and reporter bias may exist. However, we also completed a structured interview for each subject in order to minimize the possible bias. Third, PASP were measured by echocardiography instead of right heart catheterization. Although right heart catheterization is still the gold standard technique, echocardiography has been proven to be reliable for the detection of PH [42–46]. Fourth, serum thyroid autoantibody levels have not been evaluated, so that we cannot exclude the effects of autoimmunity when analyzing the association between thyroid hormones and PASP.

In conclusion, our study demonstrated that in euthyroid patients with CAD, FT_4_ was an independent risk factor for PH, and FT_4_ levels were independently associated with PASP.

## Figures and Tables

**Table 1:** Characteristics of study participants.

	PH (*n* = 86)	Non-PH (*n* = 725)	*P* value
Male (%)	69.8	78.1	0.10
PASP (mmHg)	49 ± 11	32 ± 4	<0.01
Age (years)	68.27 ± 9.42	62.74 ± 9.77	<0.01
BMI (kg/m^2^)	24.63 ± 3.19	24.63 ± 2.97	0.99
SBP (mmHg)	129.10 ± 12.25	129.02 ± 13.81	0.97
DBP (mmHg)	77.05 ± 8.23	77.42 ± 8.37	0.69
FT_4_ (mmol/L)	16.07 ± 2.55	15.30 ± 2.08	<0.01
FT_3_ (mmol/L)	4.25 ± 0.70	4.32 ± 0.56	0.35
TSH (*μ*IU/ml)	2.43 ± 1.69	2.40 ± 1.74	0.92
LVEF (%)	58 ± 14	63 ± 9	<0.01
Hypertension (%)	75.6	65.0	0.05
History of smoking (%)	16.3	7.1	0.01
ACEI & ARB (%)	54.7	47.1	0.21
Nitrates (%)	40.7	35.9	0.41
CCB (%)	26.7	26.3	1.00

PH: pulmonary hypertension; PASP: pulmonary artery systolic pressure; BMI: body mass index; SBP: systolic blood pressure; FT_4_: free thyroxine; FT_3_: free triiodothyronine; TSH: thyroid-stimulating hormone; LVEF: left ventricular ejection fraction; CCB: calcium channel blockers; ACEI & ARB: ACE inhibitors and angiotensin II receptor antagonists. Data are presented as the mean ± standard deviation or as percentages.

**Table 2:** Comparison of thyroid function parameters among subgroups with different pulmonary artery systolic pressures.

	Non-PH (*n* = 725)	Mild PH (*n* = 60)	Moderate-to-severe PH (*n* = 26)	Model 1 *P* value	Model 2 *P* value
FT_3_	4.32 ± 0.56	4.28 ± 0.68	4.18 ± 0.74	0.40	0.38
FT_4_	15.30 ± 2.07	15.75 ± 2.31^∗^	16.80 ± 2.94^∗^^#^	<0.01	0.01
TSH	2.41 ± 1.74	2.38 ± 1.78	2.53 ± 1.48	0.93	0.70

PH: pulmonary hypertension; FT_4_: free thyroxine; FT_3_: free triiodothyronine; TSH: thyroid-stimulating hormone; Model 1: unadjusted; Model 2: adjusted for age, gender, BMI, and LVEF. Data are presented as the mean ± standard deviation. ^∗^*P* < 0.05 versus the non-PH group, ^#^*P* < 0.05 versus the mild PH group.

**Table 3:** Comparison of pulmonary artery systolic pressure among the tertile groups of thyroid function parameters.

	Tertile 1	Tertile 2	Tertile 3	Model 1 *P* value	Model 2 *P* value
FT_3_	34.75 ± 8.13	32.71 ± 5.99	33.57 ± 7.50	<0.01	0.06
FT_4_	33.10 ± 5.81	33.29 ± 6.97	34.31 ± 9.23	0.14	0.42
TSH	33.05 ± 6.60	34.13 ± 8.74	33.53 ± 6.94	0.24	0.36

FT_4_: free thyroxine; FT_3_: free triiodothyronine; TSH: thyroid-stimulating hormone; Model 1: unadjusted; Model 2: adjusted for age, gender, BMI, and LVEF. FT_3_ tertiles were divided as follows: tertile 1: FT_3_ ≤ 4.1 (*n* = 308), tertile 2: 4.1 < FT_3_ ≤ 4.5 (*n* = 236), and tertile 3: FT_3_> 4.5 (*n* = 267). FT_4_ tertiles were divided as follows: tertile 1: FT_4_ ≤ 14.2 (*n* = 260), tertile 2: 14.2 < FT_4_ ≤ 16.1 (*n* = 279), and tertile 3: FT_4_> 16.1 (*n* = 272). TSH tertiles were divided as follows: tertile 1: TSH ≤ 1.57 (*n* = 275), tertile 2: 1.57 < TSH ≤ 2.59 (*n* = 277), and tertile 3: TSH> 2.59 (*n* = 259). Data are presented as the mean ± standard deviation.

**Table 4:** Multiple logistic regression analysis for pulmonary hypertension.

Independent variables	Model 1 OR (95% CI)	Model 2 OR (95% CI)	Model 3 OR (95% CI)
Gender	0.56 (0.31–0.99)	0.56 (0.32–0.99)	0.58 (0.33–1.02)
Age	1.06 (1.03–1.09)	1.06 (1.03–1.09)	1.06 (1.03–1.09)
BMI	1.01 (0.94–1.10)	1.02 (0.94–1.11)	1.02 (0.94–1.10)
SBP	0.99 (0.97–1.01)	0.99 (0.97–1.01)	0.99 (0.97–1.01)
Smoking	2.21 (1.09–4.46)	2.24 (1.11–4.55)	2.19 (1.08–4.44)
Hypertension	1.74 (0.93–3.28)	1.64 (0.87–3.10)	1.73 (0.92–3.25)
ACEI & ARB	0.96 (0.57–1.60)	0.93 (0.55–1.56)	0.94 (0.56–1.56)
CCB	0.78 (0.44–1.41)	0.76 (0.42–1.37)	0.78 (0.43–1.40)
Nitrates	1.08 (0.66–1.77)	1.07 (0.65–1.76)	1.07 (0.65–1.75)
LVEF	0.95 (0.93–0.97)	0.95 (0.93–0.97)	0.95 (0.93–0.97)
FT_3_	1.18 (0.79–1.82)	/	/
FT_4_	/	1.12 (1.01–1.25)	/
TSH	/	/	0.97 (0.84–1.12)

PH: pulmonary hypertension; BMI: body mass index; SBP: systolic blood pressure; FT_4_: free thyroxine; FT_3_: free triiodothyronine; TSH: thyroid-stimulating hormone; LVEF: left ventricular ejection fraction; CCB: calcium channel blockers; ACEI & ARB: ACE inhibitors and angiotensin II receptor antagonists. Independent variables included in the original model are as follows: model 1: FT_3_, gender, age, BMI, smoking, LVEF, SBP, history of hypertension, CCB, ACE & ARB, and nitrates; model 2: FT_4_, gender, age, BMI, smoking, LVEF, SBP, history of hypertension, CCB, ACE & ARB, and nitrates; and model 3: TSH, gender, age, BMI, smoking, LVEF, SBP, history of hypertension, CCB, ACE & ARB, and nitrates.

**Table 5:** Multiple linear regression analysis for pulmonary artery systolic pressure.

Independent variables	Model 1 *β* ± SE	Model 2 *β* ± SE	Model 3 *β* ± SE
Gender	−0.32 ± 0.67	−0.58 ± 0.65	−0.49 ± 0.65
Age	0.10 ± 0.03^∗^	0.11 ± 0.03^∗^	0.11 ± 0.03^∗^
BMI	0.03 ± 0.09	0.05 ± 0.09	0.03 ± 0.09
SBP	−0.02 ± 0.02	−0.02 ± 0.02	−0.02 ± 0.02
Smoking	1.45 ± 1.00	1.39 ± 0.99	1.42 ± 1.00
Hypertension	0.34 ± 0.67	0.32 ± 0.67	0.38 ± 0.67
ACEI & ARB	0.08 ± 0.57	0.07 ± 0.57	0.08 ± 0.57
CCB	−0.45 ± 0.67	−0.49 ± 0.67	−0.43 ± 0.67
Nitrates	−0.30 ± 0.55	−0.31 ± 0.55	−0.27 ± 0.55
LVEF	−0.14 ± 0.03^∗^	−0.13 ± 0.03^∗^	−0.14 ± 0.03^∗^
FT_3_	−0.53 ± 0.49	/	/
FT_4_	/	0.27 ± 0.12^∗^	/
TSH	/	/	0.00 ± 0.15

PASP: pulmonary artery systolic pressure; BMI: body mass index; SBP: systolic blood pressure; FT_4_: free thyroxine; FT_3_: free triiodothyronine; TSH: thyroid-stimulating hormone; LVEF: left ventricular ejection fraction; CCB: calcium channel blockers; ACEI & ARB: ACE inhibitors and angiotensin II receptor antagonists. Independent variables included in the original model are as follows: model 1: FT_3_, gender, age, BMI, smoking, LVEF, SBP, history of hypertension, CCB, ACE & ARB, and nitrates; model 2: FT_4_, gender, age, BMI, smoking, LVEF, SBP, history of hypertension, CCB, ACE & ARB, and nitrates; and model 3: TSH, gender, age, BMI, smoking, LVEF, SBP, history of hypertension, CCB, ACE & ARB, and nitrates. ^∗^*P* < 0.05.
